# Supplementary material for: MetaQTL: a package of new computational methods for the meta-analysis of QTL mapping experiments
Source: BMC Bioinformatics. 2007 Feb 8;8:49. doi: 10.1186/1471-2105-8-49 (PMC1808479; doi:10.1186/1471-2105-8-49)
Supplement: Additional File 7 — MetaQTL Package : jar file and tutorial. This Zip archive contains both the MetaQTL JAR file and the files of the tutorial. [file 1471-2105-8-49-S7.zip › org.inra.metaqtl/doc/org/metaqtl/factory/MapDBFactory.html]

MapDBFactory


|  |  |  |
| --- | --- | --- |
| **Overview** \| **Package** \| **Class** \| **Use** \| **Tree** \| **Deprecated** \| **Index** \| **Help** | |  |
| **PREV CLASS**   **NEXT CLASS** | **FRAMES**    **NO FRAMES**     **All Classes** |  |
| SUMMARY: NESTED \| FIELD \| CONSTR \| METHOD | DETAIL: FIELD \| CONSTR \| METHOD |  |


---


## org.metaqtl.factory Class MapDBFactory

```
java.lang.Object
  org.metaqtl.factory.MapDBFactory
```

---

``` public final class MapDBFactory extends java.lang.Object ```

A factory to read the list and the properties of a set
of genetic maps.

---

**Constructor Summary**

`MapDBFactory()`


| **Method Summary** | |
| --- | --- |
| `static IBioGenome[]` | `read(java.io.Reader reader)`             This method reads a file formated as follows : map property1 property2 property3 ... |

**Methods inherited from class java.lang.Object**

`clone, equals, finalize, getClass, hashCode, notify, notifyAll, toString, wait, wait, wait`

**Constructor Detail**

### MapDBFactory

```
public MapDBFactory()
```


**Method Detail**

### read

```
public static IBioGenome[] read(java.io.Reader reader)
                         throws java.io.IOException
```

:   This method reads a file formated as follows :
    map property1 property2 property3 ...
    when the fields are assumed to not contain blank space.
    For each row of the input file (except the header which
    is mandatory) a IBioGenome is created and the array
    returns should have the same length as the number of
    rows in the file.

    :   **Parameters:**: `file` - the file location. **Returns:**: the array of genetic maps. **Throws:**: `java.io.IOException`


---


|  |  |  |
| --- | --- | --- |
| **Overview** \| **Package** \| **Class** \| **Use** \| **Tree** \| **Deprecated** \| **Index** \| **Help** | |  |
| **PREV CLASS**   **NEXT CLASS** | **FRAMES**    **NO FRAMES**     **All Classes** |  |
| SUMMARY: NESTED \| FIELD \| CONSTR \| METHOD | DETAIL: FIELD \| CONSTR \| METHOD |  |


---
